# A Cross-Sectional Survey of Bacterial Species in Plaque from Client Owned Dogs with Healthy Gingiva, Gingivitis or Mild Periodontitis

**DOI:** 10.1371/journal.pone.0083158

**Published:** 2013-12-13

**Authors:** Ian J. Davis, Corrin Wallis, Oliver Deusch, Alison Colyer, Lisa Milella, Nick Loman, Stephen Harris

**Affiliations:** 1 The Waltham Centre for Pet Nutrition, Mars Petcare UK, Leicestershire, United Kingdom; 2 The Veterinary Dental Surgery, Surrey, United Kingdom; 3 Centre for Systems Biology, University of Birmingham, Birmingham, United Kingdom; University of Liverpool, United Kingdom

## Abstract

Periodontal disease is the most widespread oral disease in dogs which if left untreated results in significant pain to the pet and loss of dentition. The objective of this study was to identify bacterial species in canine plaque that are significantly associated with health, gingivitis and mild periodontitis (<25% attachment loss). In this survey subgingival plaque samples were collected from 223 dogs with healthy gingiva, gingivitis and mild periodontitis with 72 to 77 samples per health status. DNA was extracted from the plaque samples and subjected to PCR amplification of the V1-V3 region of the 16S rDNA. Pyrosequencing of the PCR amplicons identified a total of 274 operational taxonomic units after bioinformatic and statistical analysis. *Porphyromonas* was the most abundant genus in all disease stages, particularly in health along with *Moraxella* and *Bergeyella*. *Peptostreptococcus*, *Actinomyces*, and *Peptostreptococcaceae* were the most abundant genera in mild periodontitis. Logistic regression analysis identified species from each of these genera that were significantly associated with health, gingivitis or mild periodontitis. Principal component analysis showed distinct community profiles in health and disease. The species identified show some similarities with health and periodontal disease in humans but also major differences. In contrast to human, healthy canine plaque was found to be dominated by Gram negative bacterial species whereas Gram positive anaerobic species predominate in disease. The scale of this study surpasses previously published research and enhances our understanding of the bacterial species present in canine subgingival plaque and their associations with health and early periodontal disease.

## Introduction

Periodontal disease (PD) is the most widespread oral disease in dogs. Studies have demonstrated between 44% and 63.6% of dogs are affected [1-4]. Variation in prevalence estimates are likely to be due to the different age and breed compositions of the study groups and the criteria used to establish diagnosis of periodontal disease. 

It is accepted that bacteria present in human dental plaque are the aetiological agent of periodontal disease; though the specific organisms involved in the initiation of disease and the basis of the subsequent events thereafter are unclear [5]. A working hypothesis is that specific antigens or enzymes produced by bacteria in the plaque biofilm initiate activation of the host inflammatory response, the latter being the main pathological agent of periodontal disease. 

The initial stages of disease are observed clinically as red and inflamed gums, defined as gingivitis. Without treatment by removal of the plaque biofilm, gingivitis may progress to early periodontitis. The earliest stage of periodontitis (PD1) is characterised by initial tissue breakdown and loss of up to 25% attachment of the periodontal ligament surrounding the tooth root [6]. In humans this switch from gingivitis to periodontitis appears to be restricted to 10-15% of the population [7]. The onset of periodontitis is defined by irreversible tissue destruction and if left untreated will progress to extreme periodontitis (PD3-PD4). This is characterised by extensive (>50%) destruction of the periodontal ligament, gum recession and breakdown of supporting tissues eventually leading to the loss of the tooth. The periodontal disease process can be inhibited in the early stages (PD1) by dental scaling and polishing of the periodontal pocket to remove the source of inflammation (dental plaque) with subsequent regular plaque removal by tooth brushing. As such this work is focused on understanding the early stages of disease, (gingivitis through to PD1) in pet dogs where non-surgical interventions may be effective.

The diversity of bacterial species found in the canine oral microbiome has been reported using culture independent molecular methods from 51 dogs [8]. Based on full length 16S rDNA Sanger sequencing 353 taxa were identified; of these 80% were novel and only 16.4% were shared with the human oral microbiome. Not only does this indicate clear differences between the bacterial populations in human versus canine mouths but also the under representation of 16S rDNA sequences from canine oral species in public DNA sequence databases. 

A survey of the canine oral microbiota of pooled samples from gums, tongue, teeth and cheeks, of 6 clinically healthy dogs has been completed using pyrosequencing methods [9]. This approach enabled the estimation of the relative abundance of genera with the most common being *Porphyromonas* (39.2% of sequences), *Fusobacterium* (4.5%) and *Capnocytophaga* (3.8%). Significant differences in bacterial populations by oral health status have also been reported in a study of dogs with healthy mouths, gingivitis or periodontal disease (n=3 per health state; [10]). The study used culture dependant and independent approaches with identification of taxa via Sanger sequencing of 16S rDNA. Significant associations of specific bacterial taxa with disease stages were not observed due to the relatively small population size and low sequence depth (208 16S rRNA clones). However, in the human field two recent studies have demonstrated it is possible to identify species significantly more abundant in severe periodontal disease versus oral health by using larger cohorts (N>10) and the greater DNA sequence depth provided by pyrosequencing technology [11,12].

The aim of this work was to survey the oral microbiota of a sufficiently large canine cohort, at great enough depth to identify significant changes in bacterial taxa (phyla, genera and species) between dogs with healthy gingiva and those with gingivitis or mild periodontitis (PD1).

## Materials and Methods

### Ethics Statement, Sampling Strategy and Study Cohort

The study was approved by the WALTHAM^®^ Centre for Pet Nutrition ethical review committee, owner consent was obtained and an owner survey was completed for all dogs included in the study. The study cohort comprised client owned pet dogs presented at a veterinary referral dental clinic (The Veterinary Dental Surgery, Surrey, UK). Only dogs under anaesthetic for routine dental treatment or treatment for non-periodontal complications e.g. fractured teeth or other non-infectious conditions were screened for inclusion in the study. No dogs were anaesthetised solely for the collection of plaque samples. 

Dental assessments, scoring and subgingival plaque sampling were performed by a single veterinary dentist (L. Milella) to avoid variation in scoring. The periodontal health status of each dog was obtained following the Wiggs & Lobprise scoring system [6] and plaque samples taken from dogs regarded as having healthy teeth and gums, gingivitis or mild periodontitis ( <25% attachment loss). Dogs were excluded from the study if they had: 1) Significant veterinary oral care within the preceding 3 months; 2) Regular dental care at home i.e. dogs whose teeth are regularly brushed; 3) Systemic or oral antibiotic treatment at any time during the previous 3 months and 4) Evidence of any extra-oral bacterial infections in the past month. Veterinary observations suggest certain breeds may exhibit an atypical early onset/ aggressive form of periodontitis, though no data exists to confirm this these breeds (Greyhounds, Yorkshire Terriers, Maltese and Toy Poodles) were also excluded. 

Sub-gingival plaque samples were collected using a sterile periodontal probe and placed in 350µl TE buffer (50 mM Tris pH 7.6, 1 mM EDTA pH 8.0 & 0.5% Tween 20) prior to storage at -20°C. 

Healthy dogs were sampled subgingivally at eighteen sites, targeting the teeth believed to be most often affected by PD (upper 103-108 bilaterally and lower 404, 408 and 409 bilaterally), to support plaque volumes in the absence of periodontal pockets. Periodontally diseased dogs were sampled for subgingival plaque at up to twelve diseased sites (103, 104, 108, 404, 408, 409 bilaterally) during their normal periodontal treatment. In a minority of cases if a dog had sites of periodontal disease not on those teeth but more than 6 teeth were affected, the samples were taken from the affected teeth. Information on dog age, breed, size and sex was collated.

### DNA extraction & Amplification of 16S rDNA

DNA was extracted from the plaque samples using an Epicentre Masterpure Gram Positive DNA Purification Kit, according to the manufacturer’s instructions with an additional overnight lysis. Plaque samples were centrifuged at 5000 x g for 10 minutes and the cell pellet resuspended in 150μl of TE buffer. Following vortexing, 1 μl Ready-Lyse Lysozyme (Epicentre, UK) was added and the lysis mix incubated overnight at 37°C for 18hrs overnight. After DNA extraction the DNA pellet was suspended in TE buffer (10mM Tris-Cl and 0.5 mM pH 9.0 EDTA) and quantified and the purity ascertained using a NanoDrop ND1000 spectrophotometer (NanoDrop Technologies Inc). 

The V1-V3 region of the 16S rDNA was amplified from subgingival plaque DNA extractions using Extensor Hi-Fidelity PCR Enzyme Mix (AB-0792, Thermo, UK) in a 96-well format. A mix of two universal forward primers was used; FLX_27FYM (CGTATCGCCTCCCTCGCGCCATCAG **AGAGTTTGATYMTGGCTCAG** ) at 9.5pmol/μl and FLX_27F_Bif (CGTATCGCCTCCCTCGCGCCATCAG **AGGGTTCGATTCTGGCTCAG** ) at 0.5pmol/μl (where italics represent FLX Titanium Primer A and bold represents 16S sDNA primer sequence). The latter was included to ensure representation of the genus *Bifidobacter*, a lower concentration was chosen due to the low representation of this genus in previous studies of canine plaque. The DNA was to be sequenced from the reverse primer thus 20 different 7mer MID tags were included in the reverse primer (*CTATGCGCCTTGCCAGCCCGCTCAGX*XXXXXX**TYACCGCGGCTGCTGG**) where italics represent FLX Titanium Primer B, X represents MID sequence and bold represents 16S sDNA reverse primer sequence.

### Library preparation

Library preparation and sequencing was performed by Beckman Coulter Genomics, UK. The 16S rDNA amplicons were purified with Agencourt AMPure XP beads (Beckman Coulter Inc, UK), quantified using the Quant-iT™ PicoGreen® dsDNA Assay Kit (Invitrogen, UK) then pooled into groups of 20 samples prior to Emulsion PCR. Libraries were then sequenced on a Roche Genome Sequencer FLX Titanium System™ using the FLX Titanium B primer only with a target of ~ 15,000 unidirectional reads per sample.

### Sequence processing and analysis

The standard flowgram files (SFF) for each of the 223 samples were initially filtered by selecting reads with at least 360 flows and truncating long reads to 720 flows. Reads were filtered and denoised using the AmpliconNoise software (version V1.21; [13,14]). For the initial filtering step, reads were truncated when flow signals dropped below 0.7, indicative of poor quality. A maximum of 20,000 reads per sample were used with exception of a few samples due to the computational demands of the denoising algorithm. Subsequently, reads were denoised in three stages; 1) Pyronoise to remove noise from flowgrams resulting from 454 sequencing errors (PyronoiseM parameters -s 60, -c 0.01), 2) Seqnoise to remove errors resulting from PCR amplification (SeqNoiseM parameters -s 25, -c 0.08), 3) Perseus to detect and remove chimeras resulting from PCR recombination. The denoised sequences were then clustered using QIIME, a pipeline for performing microbial community analysis that integrates many third party tools which have become standard in the field. The QIIME script pick_otus.py, which utilises the Uclust software program, was used to cluster sequences with >98% identity [15]. Uclust was run with modified parameters, with gap opening penalty set to 2.0 and gap extension penalty set to 1.0 and –A flag to ensure optimum alignment [15]

Representative sequences of all observed OTUs that passed the filtering criteria for sequence abundance (see statistical analysis section) across health states were searched against the Canine Oral Microbiome Database (COMD) using BLASTN of NCBI-BLAST 2.2.27+ [16]. The COMD sequence database contained 460 published 16S sequences obtained from canine oral taxa (Genbank accession numbers JN713151-JN713566 & KF030193-KF030235; [8]). Additionally, representative sequences were searched against the 376,437 sequences in the Silva SSU database release 108 [17]. For each representative sequence the best BLAST hit in the COMD database was chosen as the reference sequence. If the alignment did not meet the cut-off criteria of ≥98% sequence identity and ≥98% sequence coverage the best hit from the Silva database was chosen. The assignments were checked for redundancies (two or more OTUs assigned to the same reference sequence). Redundancies were resolved by keeping the taxonomy for the OTU with the better match and assigning the next best match to the other OTU.

A multiple sequence alignment (MSA) was constructed by aligning each reference sequence to the Greengenes [18] core set (revision 3rd May 2011) with PyNAST [19] using the script align_seqs.py of the Qiime pipeline [15]. The MSA was filtered using the filter_alignment.py script of the Qiime pipeline. The MSA was converted to Phylip interleaved format using ClustalW 2.1 [20]. A maximum likelihood tree of 1000 bootstrap replicates was inferred with PhyML 3 revision 20120412 [21]. A GTR model of nucleotide substitution was chosen and the proportion of invariant sites was estimated from the data. Evolutionary rates were approximated by a discrete gamma model of eight categories. The tree was visualised and combined with abundance and significance data in iTOL [22,23]. The resulting tree is supplied as Figure S1.

A second tree with a reduced amount of taxa was inferred at the genus level. For this purpose all species of the same genus were collated into a single taxon. The 16S sequence of the most abundant species of that genus was used for tree inference using the methods described above. If no genus information was present, taxa forming a clade in the full tree were grouped together and the new taxon was named e.g. “Actinomyces clade A”. Abundance information was added up for all members of each summarised taxon and plotted on the tree using iTOL. The tree was complemented with information on the number of original taxa summarised and the number of significant taxa. See Table S1 for information on which taxa were grouped together.

### Statistical analysis

Health and disease associations: OTUs were classified in a single group of “rare” taxa if either they were present in each health status group at an average proportion below 0.05% or were present in less than two samples. The 0.05% cut-off was selected based on statistical analysis of data from mock communities containing 17 known species sequenced on five separate 454 runs. The mock communities were analysed for presence and absence of species using a false positive rate of 0.3% (i.e. finding species that were not included in the mock community) and false negative rate of 1.7% (i.e. the failure to identify the species that were known to be present) and aimed for a coefficient of variation of <20% (data not shown). The most abundant OTUs were then analysed using logistic regression analyses (Generalised linear model with a binomial distribution and logit link) for proportions, using the count of an OTU out of the total number of sequences, with health status included as a fixed effect. As the data were of very low proportions ~0.1%, a permutation test (1000 permutations) was used to allow for deviations from the logistic regression analysis assumptions. The permutation test p-values were then adjusted according to the false discovery method of Benjamini and Hochberg [24] to allow for the increased likelihood of false positives when analysing the 274 OTUs.

Principal component analysis (PCA) was performed on the log_10_ (proportions+0.00003 to allow for zeros) to determine if variability of the most abundant OTUs was associated with health status, gender, size or age.

Gram-stain status: The OTUs excluding rares were classified as Gram positive or Gram negative based on literature searches using the genus name. The number of Gram positive sequences, out of the total number of sequences, were then analysed by logistic regression for proportions (allowing for estimation of over dispersion) with health status as a fixed effect.

Oxygen Requirement: The non-rare OTUs were classified as aerobic, anaerobic or facultative anaerobic based on literature searches using the genus name. The number of aerobic, anaerobic and facultative anaerobic sequences, out of the total number of sequences, were then analysed (separately) by logistic regression for proportions (allowing for estimation of over dispersion) with health status as a fixed effect.

Shannon diversity index: a general linear model was used to analyse the indexes, with health status as a fixed effect and weighting by health status variability (to allow for significant differences in the variability of indexes between health statuses).

Species richness: a linear model was used to analyse the number of OTUs identified (including the rare sequences), with health status as a fixed effect, the total number of sequences as a covariate (to adjust for the differing number of sequences between samples) and weighting by health status variability (to allow for significant differences in the variability of the number of OTUs between health statuses). 

Univariate statistical analyses were performed in GenStat v14.1 software and multivariate analyses in R v3.0.1. 

## Results

### Study Cohort

Subgingival plaque bacterial communities were sampled from a total of 223 dogs; 72 with healthy gingiva, 77 with gingivitis and 74 with mild periodontitis (PD1). Dog size and age are putative risk factors for periodontitis and therefore sample associated metadata were also obtained (see Table 1). The majority of samples were collected from small, medium and large dogs with giant dogs represented at a much lower frequency. As expected the mean age of dogs increased with disease stage and significant differences (*p*<0.001) were observed in the mean ages of dogs in health compared to gingivitis and gingivitis compared to mild periodontitis using a two-tailed *t*-test with unequal variance. Lesser significance was observed in health versus gingivitis (*p*<0.05).

**Table 1:** Summary of metadata for sample cohorts, numbers shown are mean ± s.d.

	**Health**	**Gingivitis**	**Mild periodontitis**
**Age**	4.5 ± 2.3 years	5.0 ± 2.8 years	7.3 ± 3.1 years
**Gender**	31 female, 41 male	38 female, 39 male	32 female, 42 male
**Size**	8 small, 30 medium, 29 large, 3 giant, 2 unknown	12 small, 31 medium, 30 large, 3 giant, 1 unknown	23 small, 16 medium, 31 large, 3 giant, I unknown
**Breed**	57 pure breed, 15 cross breeds	69 pure breed, 8 cross breeds	61 pure breed, 13 cross breeds

Dog size were summarised as small (<10kg) medium (10-25kg), large (>25-45kg) and giant (>45kg).

### Sequence quality

The 223 canine subgingival plaque samples were analysed by 454-pyrosequenicng of the 3’ end of the V1-V3 region and a total of 6,071,129 sequence reads were obtained that passed the sequencing provider’s initial sequence quality filter. After Pyronoise, Seqnoise and chimera removal using Perseus the number of sequence reads was reduced to 3,110,837. The final number of sequence reads per sample ranged from 2,801 to 30,050 with a median number of reads of 11,682, 12,674 and 15,111 from healthy, gingivitis and mild periodontitis samples respectively.

### Bacterial composition of canine plaque

The resulting 3,110,837 sequences were assigned to 6,431 operational taxonomic units (OTUs) using U-Clust within QIIME and a cut-off of ≥98% sequence identity. OTUs were classed and grouped as rare if either they were present in each health status group at an average proportion below 0.05% or were present in less than two samples (see methods for rationale). This reduced the number of OTUs analysed to 274 plus the rare group. 

Taxonomic assignment of each of the 274 OTUs resulted in 222 (81%) and 30 (11%) mapping to sequences within COMD [8] and Silva respectively with ≥98% identity. The remaining 22 OTUs (8%) shared between 91.4% and 97.7% identity to sequences within the Silva database. The majority of the sequences belonged to seven phyla; Firmicutes (28.5%), Bacteroidetes (26.5%), Proteobacteria (17.36%), Actinobacteria (15.3%), Fusobacteria (3.7%), Spirochaetes (1.9%) and TM7 (1.1%). There were also a further five phyla identified; Synergistetes (0.9%), Chloroflexi (0.7%), SR1 (0.4%), Tenericutes (0.09%) Elusimicrobia (0.04%) and a small proportion of the sequences were of unknown phylogeny (0.08%). The rare group accounted for the remaining 3.4% of the sequence reads. 

**Figure 1:**
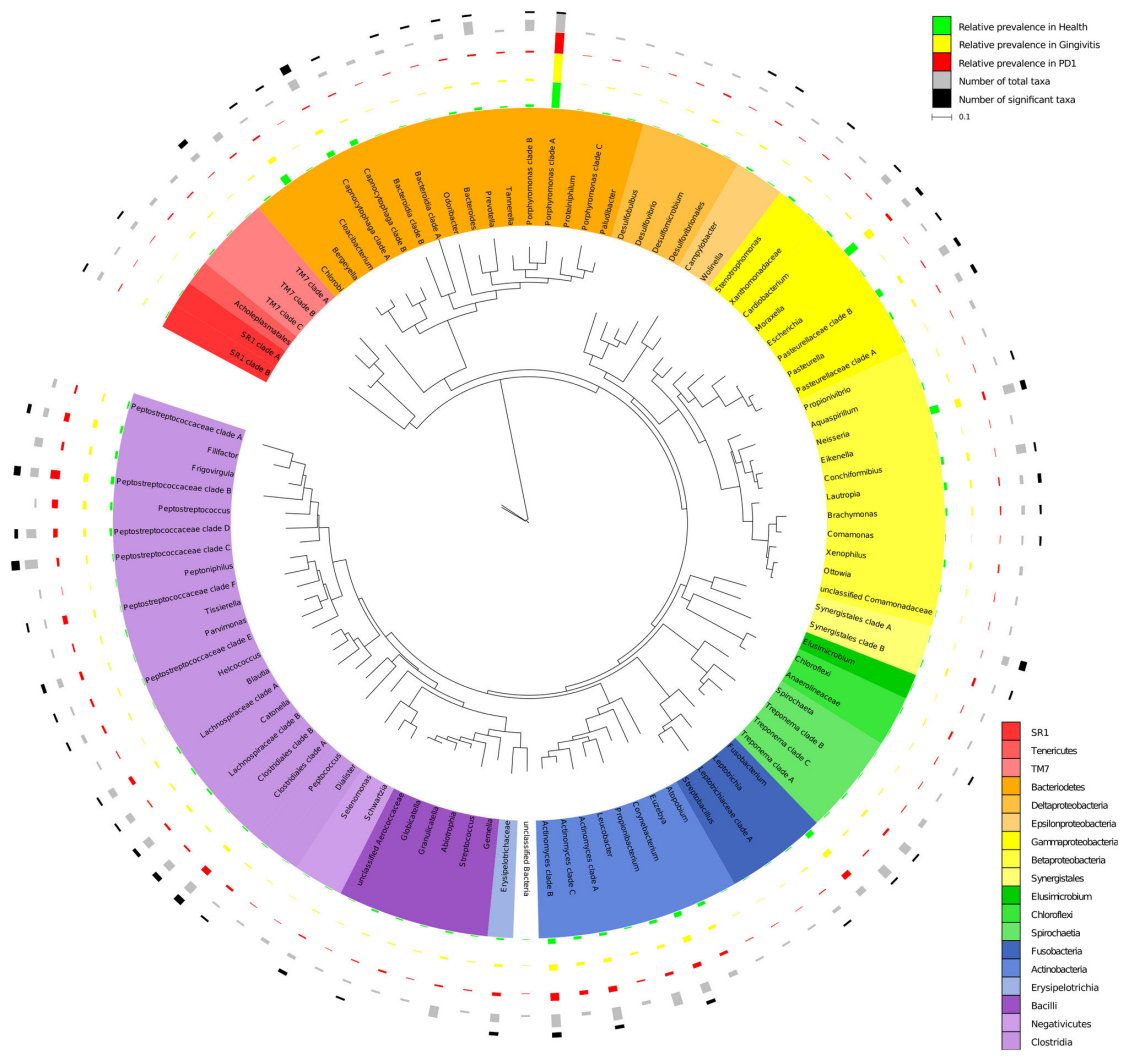
Circular maximum likelihood tree of full length 16S rRNA genes at level of genus. The inner band shows genera coloured by phylum/class (based on NCBI taxonomy) , the next three bands depict relative abundance of each genus in health (green), gingivitis (orange) and mild periodontitis (red). The two outer bands show the total number of taxa identified within the genus (grey, ranging from 1 to 11) and number of those taxa that showed a significant association with a health status (black, ranging from 0 to 5).

A phylogenetic tree inferred at the genus level is shown in Figure 1 and at the species level in Figure S1. The 99 genera observed contained 274 species, of which the 26 most abundant species accounted for approximately 50% of the sequence reads (see Table 2 and Figure S1). *Porphyromonas cangingivalis* COT-109 (OTU# 2517) was the most abundant taxa representing 7.4% of the total number of sequence reads. *Moraxella* sp. COT-396 (4266) and *Actinomyces canis* COT-409 (OTU# 6029) were the next most abundant representing 3.47% and 3.23% of the sequence reads respectively. Five other species each represented between 2% and 2.8% of the population; *Bergeyella zoohelcum* COT-186 (OTU# 2232), *Peptostreptococcus* sp. COT-033 (OTU# 6027), *Peptostreptococcaceae* sp. COT-004 (OTU# 5570)*, Porphyromonas gulae* COT-052 (OTU# 2678), *Porphyromonas gingivicanis* COT-022 (OTU# 5364). A further 18 OTUs represented between 0.85% and 2% of the population and the remaining 248 OTUs ranged in relative abundance from 0.01% to 0.81%

**Table 2:** The 26 most abundant operational taxonomic units (OTUs) in canine plaque from dogs with healthy gingiva, gingivitis and mild periodontitis.

**OTU**	**Species**	**Percentage identity**	**Total number of sequence reads**	**Proportion of total sequence reads (%)**
2517	*Porphyromonas cangingivalis* COT-109	99.4	230327	7.40%
4266	*Moraxella* sp. COT-396	98.9	107867	3.47%
6029	*Actinomyces canis* COT-409	99.1	100436	3.23%
2232	*Bergeyella zoohelcum* COT-186	99.1	87570	2.81%
6027	*Peptostreptococcus* sp. COT-033	99.7	74661	2.40%
5570	*Peptostreptococcaceae* sp. COT-004	100.0	65764	2.11%
2678	*Porphyromonas gulae* COT-052	100.0	64382	2.07%
5364	*Porphyromonas gingivicanis* COT-022	99.7	63838	2.05%
2908	*Filifactor villosus* COT-031	100.0	60684	1.95%
2905	*Actinomyces* sp. COT-083	100.0	60238	1.94%
3307	*Actinomyces* sp. COT-252	100.0	56776	1.83%
2233	*Neisseria shayeganii* COT-090	100.0	52354	1.68%
5572	*Fusobacterium* sp. COT-189	99.1	50612	1.63%
3434	*Porphyromonas canoris* COT-108	100.0	47457	1.53%
3638	*Porphyromonas gulae* COT-052	99.7	46699	1.50%
2576	*Corynebacterium freiburgense* COT-403	99.7	41549	1.34%
2463	*Peptostreptococcaceae* sp. COT-077	100.0	39940	1.28%
1916	*Clostridiales* sp. COT-028	100.0	39516	1.27%
4116	*Fusobacterium* sp. COT-169	99.4	39001	1.25%
1678	*Pasteurellaceae* sp. COT-080	100.0	37073	1.19%
4929	*Capnocytophaga* sp. COT-339	100.0	36692	1.18%
5804	*Erysipelotrichaceae* sp. COT-311	100.0	31319	1.01%
368	*Peptostreptococcaceae* *sp.* COT-135	100.0	31151	1.00%
6025	*Lachnospiraceae* sp. COT-036	100.0	29757	0.96%
1773	*Moraxella* sp. COT-018	100.0	27348	0.88%
5514	*Capnocytophaga cynodegmi* COT-254	100.0	26402	0.85%

### Associations with health and disease

**Figure 2:**
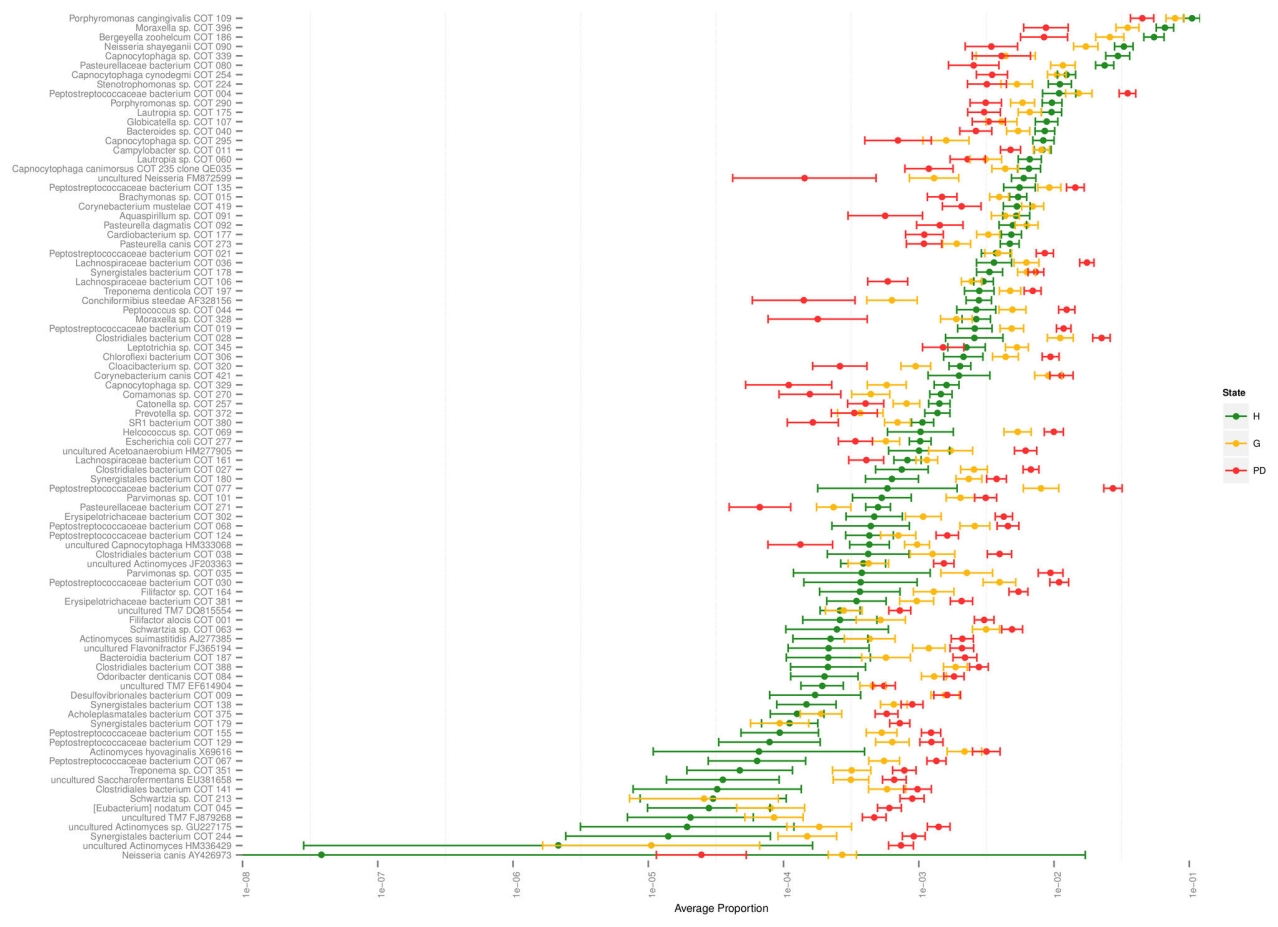
Average proportions with 95% confidence intervals for OTUs with a significant health status effect; health (green), gingivitis (orange) and mild periodontitis (red).

Logistic regression analysis identified that health status had a statistically significant effect on 90 of the 274 OTUs after randomisation and multiplicity correction. Of these, 54 showed a significant difference between health and gingivitis, 73 showed a significant difference between gingivitis and mild periodontitis and 87 showed a significant difference between health and mild periodontitis (see Figure S1, Figure 2 and Table S2). 

Of the most abundant health associated species, *Moraxella* sp. COT-396 (QIIME OTU# 4266, 6.61%), *Bergeyella zoohelcum* COT-186 (OTU# 2232, 5.48%), *Neisseria shayeganii* COT-090 (OTU# 2233, 3.28%) and *Pasteurellaceae* sp. COT-080 (OTU# 1678, 2.37%) were significantly more abundant in health and gingivitis than in mild periodontitis and were also significantly more abundant in health than gingivitis (See Table S2). *Capnocytophaga* sp. COT-339 (OTU# 4929, 2.96%), *Stenotrophomonas* sp. COT-224 (OTU# 564, 1.1%) were also significantly more abundant in health than in gingivitis and mild periodontitis but the relative abundance in gingivitis and mild periodontitis were not significantly different. Again, *Porphyromonas cangingivalis* COT-109 (OTU# 2517, 10.47%) and *Capnocytophaga cynodegmi* COT-254 (OTU# 5514, 1.24%) were significantly more abundant in health and gingivitis than in mild periodontitis but the relative abundance in health and gingivitis did not significantly differ.

The most abundant disease associated species included *Peptostreptococcaceae* sp. COT-004 (OTU# 5570, 3.5%) and *Lachnospiraceae* sp. COT-036 (OTU# 6025, 1.7%) which were significantly more abundant in mild periodontitis than health and gingivitis and did not significantly differ in their relative abundance in health and gingivitis. *Clostridiales* sp. COT-028 (OTU# 1916, 2.2%), *Peptostreptococcaceae* sp. COT-135 (OTU# 368, 1.4%), *Peptostreptococcaceae* sp. COT-077 (OTU# 2463, 2.7%), *Peptococcus* sp. COT-044 (OTU# 4905, 1.2%), *Peptostreptococcaceae* sp. COT-019 (OTU# 908, 1.2%) and *Peptostreptococcaceae* sp. COT-030 (OTU#4774, 1.1%) were also significantly more abundant in mild periodontitis than in health and gingivitis but were also more abundant in gingivitis than health. *Corynebacterium canis* COT-421 (OTU# 149, 1.1%) was significantly more abundant in mild periodontitis and gingivitis than health but gingivitis and mild periodontitis samples were not significantly different.

**Figure 3:**
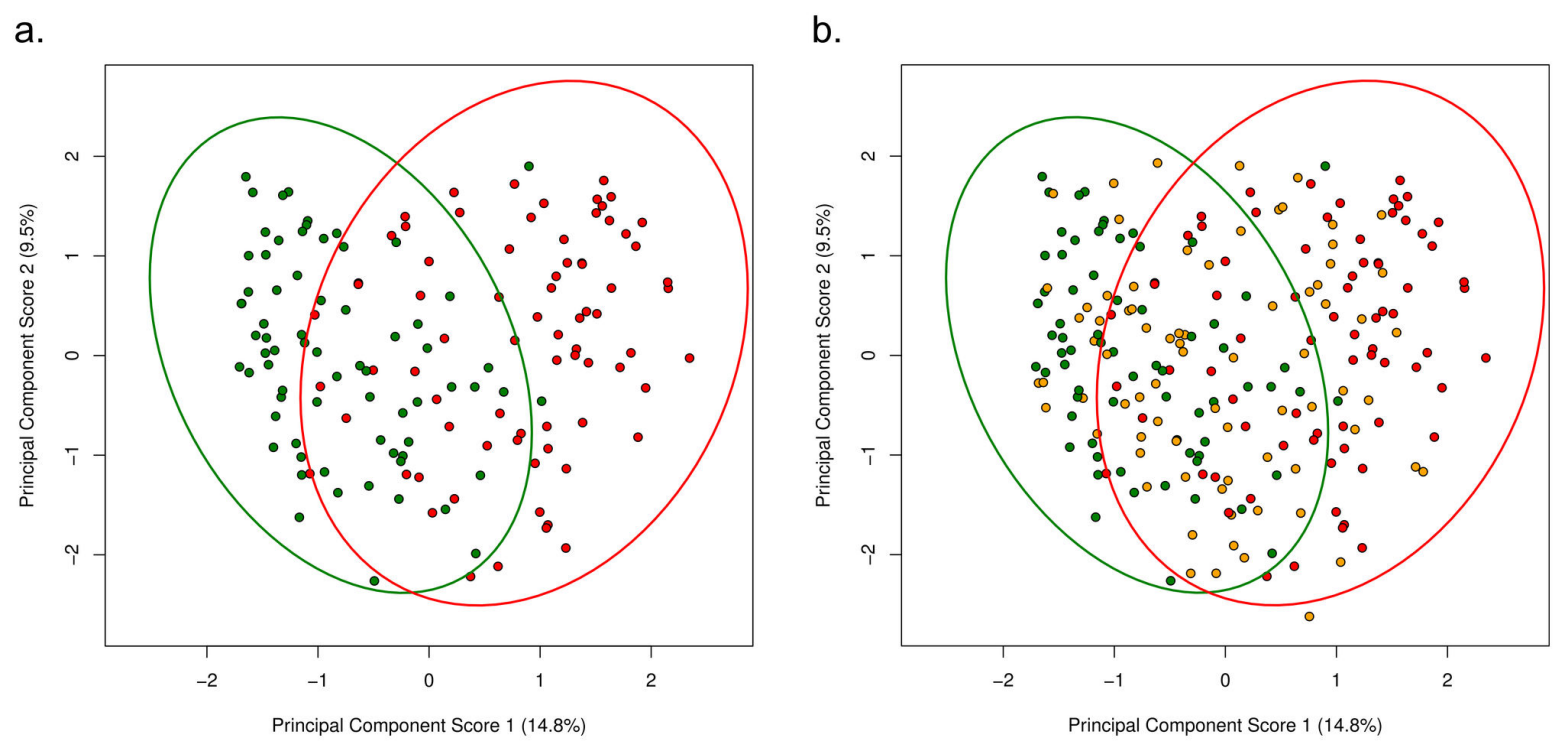
A & B: Principal component scores from analysis performed on the log_10_ proportions of OTUs identified in each individual coloured by health status; health (green), gingivitis (orange) and PD1 (red). The 95% confidence ellipses for the scores were calculated by the R package vegan.

Principal component analysis was used to investigate correlations of OTUs with health status, age, size and gender. The first component explained 14.7% and the second component 9.5% of the variability in the OTU log_10_ proportions (see Figure 3a). Discrete clustering of healthy and mild periodontitis samples was seen, whilst gingivitis samples overlaid both health and mild periodontitis clusters (see Figure 3b). Gender, size and age did not appear to show any distinct clusters (data not shown).

### Gram-stain status and oxygen requirements

**Figure 4:**
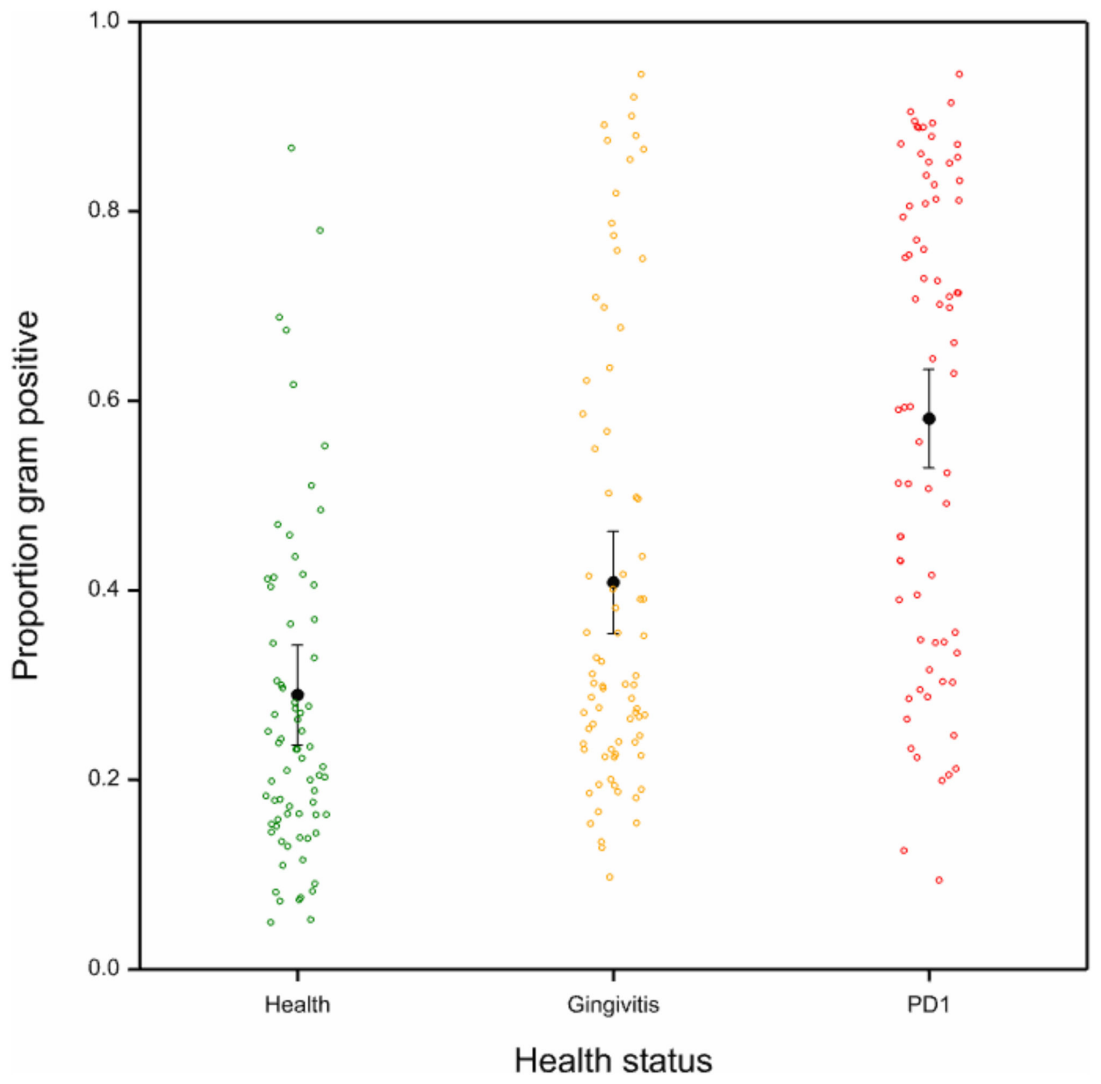
Proportions of Gram positive OTUs for each sample by health status; health (green), gingivitis (orange) and mild periodontitis (red). Black bars indicate mean proportion of OTUs that are Gram positive with 95% confidence intervals.

The probable Gram-stain status was determined by literature searches followed by logistic regression analysis of proportions of Gram positive or Gram negative non-rare OTUs; this showed that health, gingivitis and mild periodontitis groups were significantly different. Samples from dogs with mild periodontitis had a significantly higher proportion of Gram positive OTUs than those from dogs with gingivitis (*p*<0.001) and healthy gingiva (*p*<0.001). Gingivitis samples had a significantly higher proportion of Gram positive OTUs than samples from the healthy group (*p*=0.003; see Figure 4). These data show that plaque samples from dogs with mild periodontitis have a higher proportion of Gram positive species whereas those isolated from healthy gingiva are dominated by Gram negatives.

**Figure 5:**
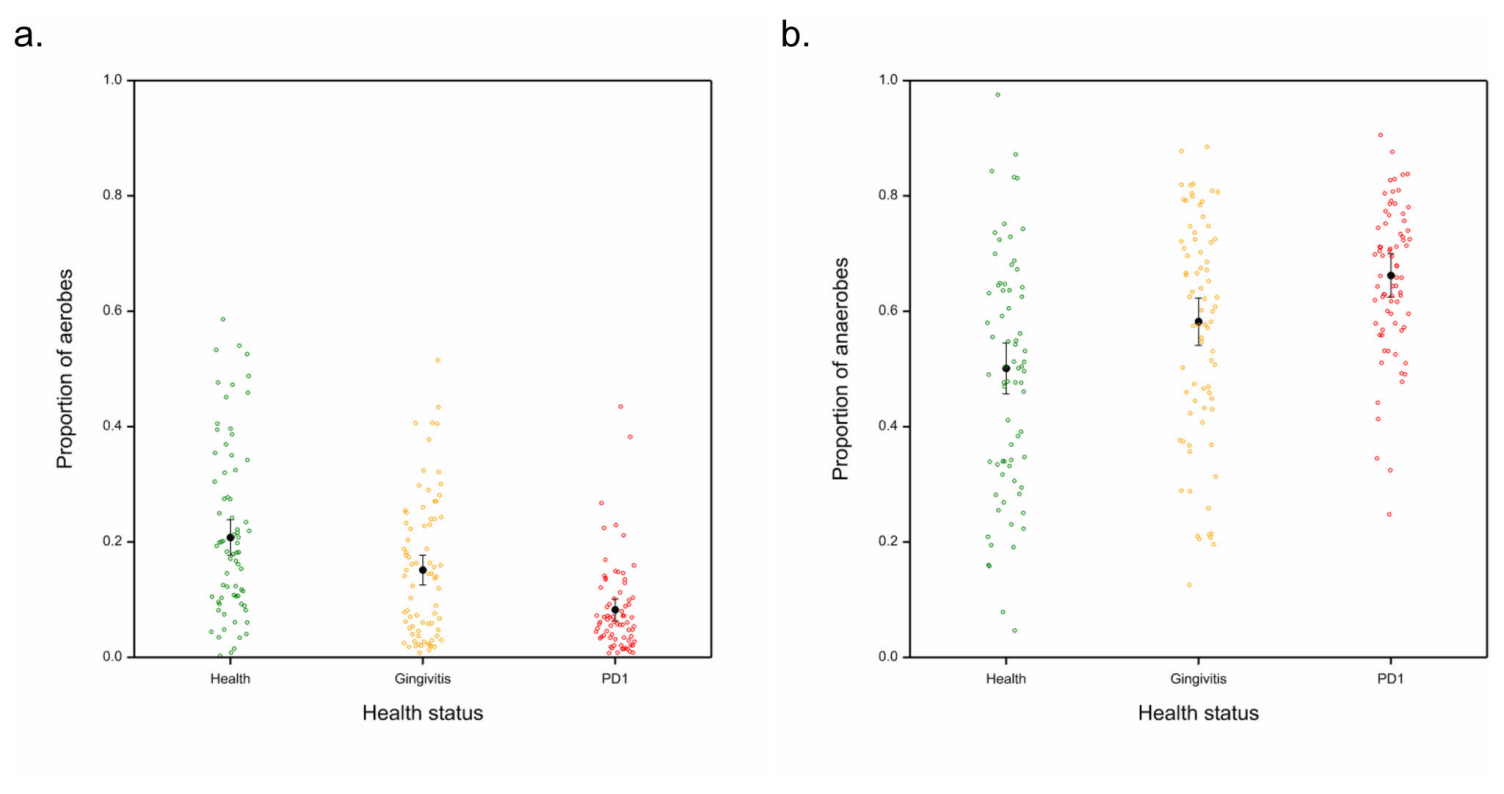
Proportions of A) aerobes & B) anaerobes for each sample by health status; health (green), gingivitis (orange) and mild periodontitis (red). Black bars indicate mean proportion of species that are aerobic or anaerobic with 95% confidence intervals.

The probable oxygen requirements were also determined by literature searches and analysed by logistic regression for proportions of aerobes, facultative anaerobes and anaerobes. Clear differences in oxygen requirements were observed between the bacterial population in healthy, gingivitis and mild periodontitis samples. Samples from dogs with healthy gingiva had significantly higher proportions of aerobes than gingivitis and periodontitis samples (*p*=0.006 & *p*<0.001 respectively) and gingivitis samples had a significantly higher proportion of aerobes than samples from dogs with mild periodontitis (*p*<0.001; see Figure 5). Mild periodontitis samples had a significantly higher proportion of anaerobes than healthy and gingivitis samples (*p*<0.001 & *p*=0.005 respectively) and gingivitis samples had a significantly higher proportion than healthy samples (*p*=0.009). In terms of facultative anaerobes, healthy and gingivitis samples did not significantly differ (*p*=0.166) and the same was true for gingivitis and periodontitis samples (*p*=0.165). However, there were significantly more facultative anaerobes in health than mild periodontitis (*p*=0.006).

### Species richness and diversity

**Figure 6:**
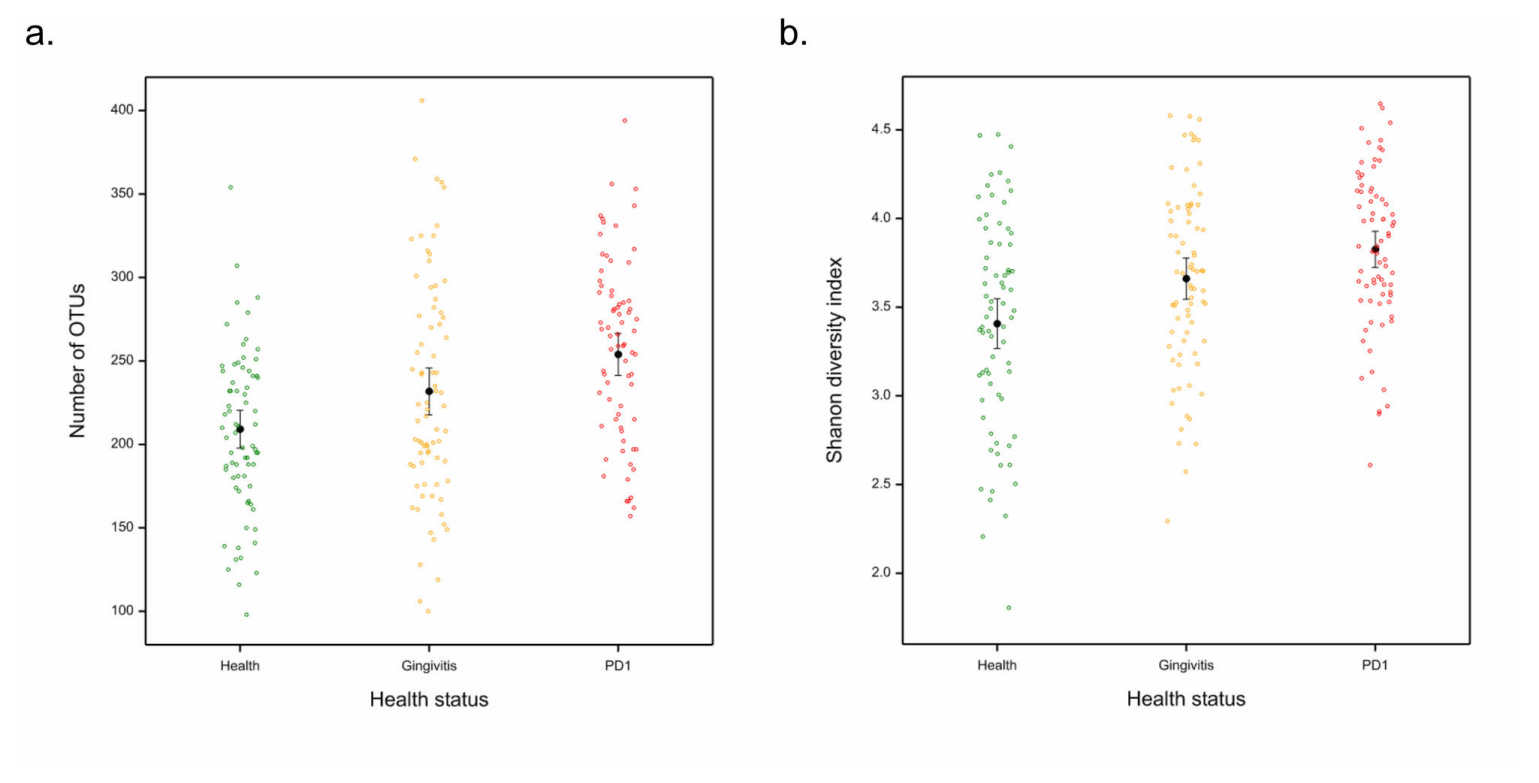
A) Number of OTUs and B) Shannon diversity Index for plaque samples from healthy dogs (green), dogs with gingivitis (orange) and those with mild periodontitis (red).

A linear model was used to compare the number of operational taxonomic units (OTUs) including rare OTUs in health, gingivitis and mild periodontitis. This showed that all health statuses were significantly different (see Figure 6). There were significantly more OTUs in plaque samples from dogs with mild periodontitis than gingivitis (*p*=0.022). In addition, samples from healthy gingiva contained significantly fewer OTUs than PD1 (*p*<0.001) and gingivitis samples (*p*=0.014).

A linear model was used to analyse the Shannon diversity index data and showed that all health statuses were significantly different (see Figure 6). The Shannon diversity index was significantly greater in PD1 samples than health samples (*p*<0.001) and also in mild periodontitis versus gingivitis samples (*p*=0.036). The diversity index was also significantly smaller in samples from healthy dogs compared with gingivitis samples (*p*=0.0065). This demonstrates that plaque samples from dogs with mild periodontitis are more species rich and diverse than those obtained from dogs with healthy gingiva.

## Discussion

 Our previous study of bacterial diversity highlighted the novelty of canine oral taxa and the importance of using a relevant database (80% were novel to current public DNA sequence databases) with which to annotate taxonomic units to species level [8]. Having the full length 16S rRNA sequence from the 282 novel canine oral taxa combined with public databases was crucial to extract meaningful information from this relatively short read 454 dataset. Since only 22 of the 274 most prevalent OTUs were novel (i.e. were not within 98% identity to known species from either the COMD or Silva databases) this indicates that the vast majority of the most prevalent species have now been found (accepting the limits of the technology used). 

Two key criteria that have influenced the results of this study are the level of abundance below which a species was deemed as rare (or sequence noise) and secondly the percentage shared identity used to group taxa at the species level. For the former, the rare or sequence noise group was defined as those sequences that either had an average prevalence in each health status of less than 0.05% or were present in less than two samples. This was supported by the statistical analysis of mock community sequences which showed that above 0.05% cut off 99.7% of species identified were genuinely present and not sequence noise. However as a consequence, some rare species or OTUs that were actually present were excluded from our analyses as individuals but were instead considered as part of the rare group. For clustering of species, the cut off of ≥98% shared identity of 16S rDNA sequence over (trimmed) sequences of between 150 and 350bp was based on the current opinion of the field [8,12]. Inherent with inferring taxonomy via 16S rRNA is the highly conserved nature of certain genera. This resulted in the Enterobacteriaceae being annotated solely as *Escherichia coli*. For both criteria the decisions are ultimately pragmatic and restricted by the technological and analytical tools at hand. 

This work is the most in depth cross sectional survey of bacterial species in canine plaque from dogs with healthy gingiva, gingivitis and mild periodontitis to-date. Previous studies of canine periodontal disease have not been able to assign significant associations of bacterial species to disease stages [10,25]. This was due to limited cohort sizes (N<6) and limited sequence depths as they were based on bacterial isolation or Sanger sequencing of 16s rDNA clones.

Parallel research on the species associated with human periodontal health and disease using similar methods offers the opportunity to better understand the polymicrobial populations involved in the aetiology of periodontal disease [11,12] by comparison. Assuming the disease pathology is conserved between human and canine (for many years human periodontal disease models have been based on dog) then the differences in observed species are most likely due to different growth conditions in the dog’s mouth, saliva and diet. Conversely some commonality in functions must remain between human and canine oral microbes as they both instigate gingivitis and periodontitis.

As a whole the most predominant phyla observed were the *Bacteroidetes, Proteobacteria, Firmicutes* and *Actinobacteria* from which 26 of the most abundant species made up 49.8% of all sequences. The proportions of these phyla shifted between disease stages with the *Proteobacteria* and *Bacteroidetes* being most abundant in plaque from the healthy cohort and the *Firmicutes* being more abundant in the mild periodontitis cohort. This is in contrast to surveys of human plaque where *Actinobacteria* were prevalent in health and higher proportions of *Spirochetes*, *Synergistetes*, *Firmicutes* & *Chloroflexi* observed in periodontitis (Abusleme et al., 2013). Comparisons with the human oral microbiota become most striking at the genus & species level. Whilst *Streptococcus* spp. are abundant in healthy humans they are rare in dogs; the lack of cariogenic *Streptococcus* spp. is presumably the reason dental caries is a rarely observed disease in dogs. Of note in this respect is the pH of canine saliva (pH 8.5, [26]), which is considerably more alkaline than that of human saliva (pH 6.5 to 7.5, [27]). It is possible that this difference in pH contributes to the lack of Streptococci in the dog oral cavity along with the lower level of sugars in the diet. The latter would be consistent with the recent observation that the human oral microflora evolved to a more cariogenic nature following the introduction of processed sugars to the diet during the industrial revolution [28]. In healthy dogs *Porphyromonas cangingivalis* Canine Oral Taxon (COT)-109, *Moraxella* sp. COT-396 and *Bergeyella zoohelcum* COT-186 were the most abundant species. The latter two are also abundant in human health but the abundance of a Porphyromonad in health in dog is in contrast to historical understanding of the human oral microbiome where *P. gingivalis* has been synonymous with the red complex and human periodontal disease [29]. The recent survey by Abusleme et al. [11], also noted a *Porphyromonas* spp. closely related to *Porphyromonas catoniae* was more prevalent in healthy human plaque versus periodontitis samples; suggesting the role of certain *Porphyromonas* spp. in periodontal disease may need to be reassessed. The abundance of *Porphyromonas, Moraxella* and *Bergeyella* in healthy dogs was also observed in a recent 454 pyrosequencing study of 6 dogs [9]. 

With respect to canine periodontal disease the *Actinomyces*, *Peptostreptococcaceae* and *Porphyromonas* species predominated. The most abundant species being *P. cangingivalis* COT-109 (again), *Peptostreptococcus* sp. COT-033, *Actinomyces* sp. COT-374, *Peptostreptococcaceae* XI [G-1] sp. COT-004 and *Peptostreptococcaceae* XIII [G-2] sp. COT-077. *Fusobacterium* and *Treponema* spp associated with human periodontal disease where present but at lower abundance and only one *Treponema* spp. (*T. denticola*) was significantly associated with mild periodontitis [11,12]. This difference in the apparent importance of Treponemes in the disease state may be as a result of an earlier stage of periodontitis being surveyed in this study relative to the human studies. It is also accentuated by the large number of different *Treponeme* species identified in our analysis (16 species- Figure S1) leading to fragmentation of the abundance. Indeed, if grouped at the genus level the *Treponeme* species make up 2.15% of the total in disease. 

Relatively few species were associated solely with gingivitis (*Leptotrichia* sp. COT-345, *Neisseria canis* AY426973 and an uncultured Capnocytophaga sp. HM333068). The abundance of health associated species did not always follow a linear reduction in abundance in gingivitis through to PD1, for many their abundance was also relatively high in gingivitis. This was also true for mild periodontitis associated species making it challenging to differentiate a health/ gingivitis associated species from a health associated species or a gingivitis/ periodontitis associated species from a periodontitis associated species. Presumably certain health associated species can compete in the gingivitis environment but not in periodontitis and vice versa for periodontitis associated species. 

In human plaque Gram-positive bacteria have traditionally been regarded as health associated and anaerobic Gram-negative bacteria as disease associated [30]. Griffen’s recent survey noted that this may be an over simplification with at least one Gram-positive bacteria (*Filifactor alocis*) being abundant in human disease [12]. Our observations in dog are in contrast to those from the human oral microbiome with Gram negative species being significantly more abundant in healthy plaque samples and Gram positives significantly more abundant in periodontitis plaque samples. The lack of Streptococci in dog results in the health associated species being dominated by Gram-negative aerobes. In contrast to health, the abundance of periodontitis associated Firmicutes, particularly *Peptostreptococcaceae* spp., means that Gram-positive anaerobes predominate in the periodontitis associated species. The environmental pressures that drive selection of species presumably include nutrient sources, oxygen stress, pH and immunological factors. We hypothesise that the major health associates may be aerobic early colonisers that are able to metabolise salivary carbohydrates such as mucins and proline rich proteins. With chronic gingivitis and periodontitis, uncontrolled inflammation and bacterial activity result in the destruction of gingival tissue leading to anaerobic periodontal pockets containing protein rich gingival crevicular fluid and immunological agents. This may then drive the rise in abundance of proteolytic anaerobic *Clostridiales* and *Peptostreptococcaceae* and *Porphyromonads* known for their ability to resist host defences and utilise host oxidative immune responses [31]. The ability of the Gram negative anaerobe *Porphyromonas cangingivalis* to predominate in all three health states suggests that it is both metabolically flexible enough to colonise in health and able to compete against other *Porphyromonas* spp. in the disease environment. It’s ability to prosper in health which is traditionally considered to be a more aerobic environment is interesting given that Porphyromonads are strict anaerobes.

Surveying the abundance of bacterial species in health, gingivitis and mild periodontitis has enabled the identification of their associations with disease stages. However these are merely associations. Which species drive the disease process as opposed to being better suited to survival in the disease environment is more difficult to elucidate. Species prevalent in health may have a role in maintaining a healthy environment or may simply have the optimum physiological and metabolic mechanisms to compete in the nutritional environment conferred in healthy gingiva. The two are not mutually exclusive and it is beyond the remit of this survey to postulate which is the case. Future genomic and phenotypic work will aid our understanding of the role of these organisms in the development of periodontal disease.

## Supporting Information

Figure S1 **Circular maximum likelihood tree of full length 16S rRNA genes at level of species.** The inner band shows species coloured by phylum/class (based on NCBI taxonomy), the next three bands depict relative abundance of each species in health (green), gingivitis (orange) and mild periodontitis (red). The outer band highlights species that showed a significant association with a health status (black).
(PDF)

Table S1 **The species that were grouped to genus/ clade level in Figure 1.** The number of species grouped into each genus is also included. See figure S1 for a tree of these species.
(DOC)

Table S2 **Summary of OTUs significantly associated with health (**H**), gingivitis (**G**) or mild periodontitis (PD1).**
(DOC)
