# Supplementary figures and images for: A Cross-Sectional Survey of Bacterial Species in Plaque from Client Owned Dogs with Healthy Gingiva, Gingivitis or Mild Periodontitis
Source: PLoS One. 2013 Dec 13;8(12):e83158. doi: 10.1371/journal.pone.0083158 (PMC3862762; doi:10.1371/journal.pone.0083158)

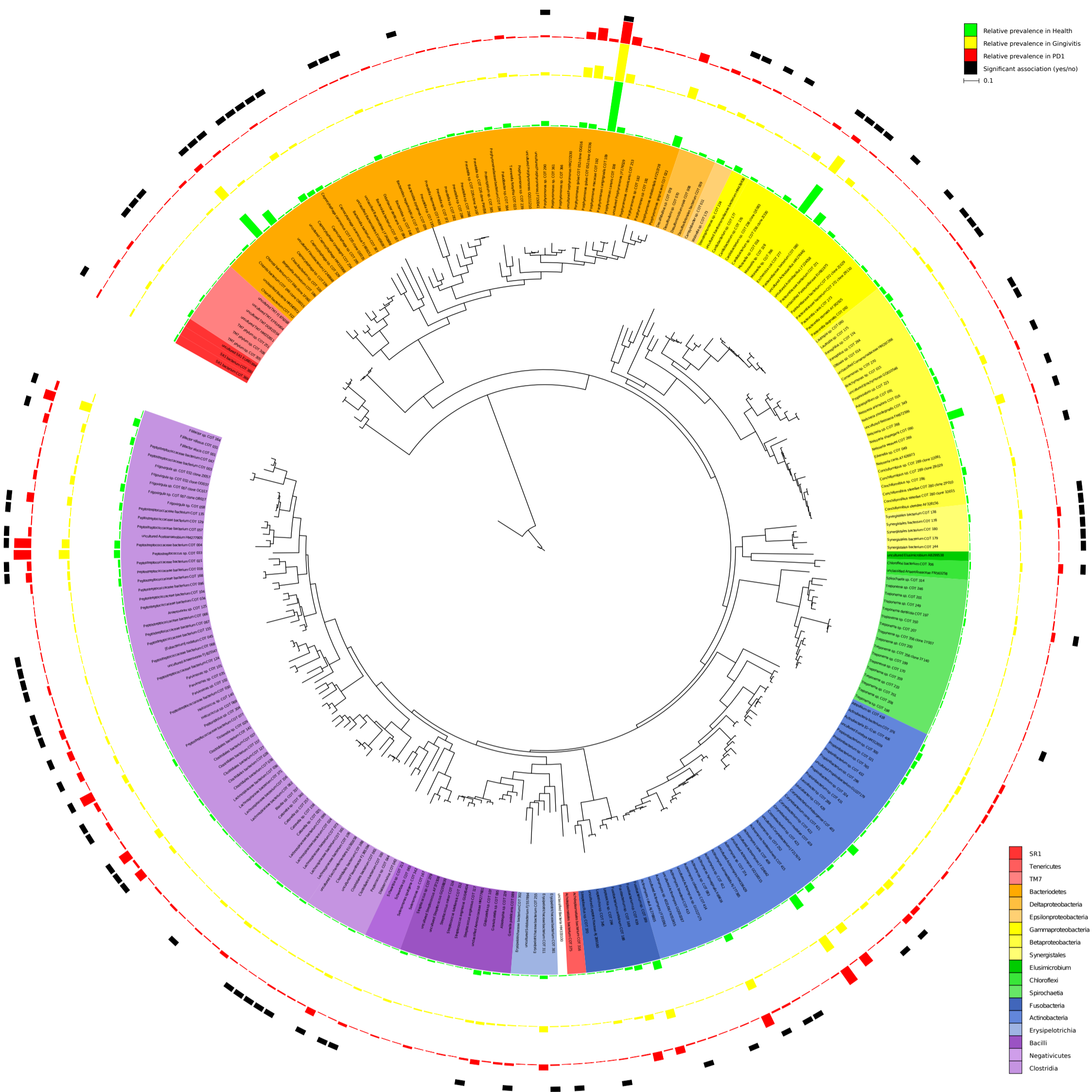

Supplement: Figure S1 — Circular maximum likelihood tree of full length 16S rRNA genes at level of species. The inner band shows species coloured by phylum/class (based on NCBI taxonomy), the next three bands depict relative abundance of each species in health (green), gingivitis (orange) and mild periodontitis (red). The outer band highlights species that showed a significant association with a health status (black). (PDF) [file pone.0083158.s001.pdf]
