# Supplementary material for: A Cross-Sectional Survey of Bacterial Species in Plaque from Client Owned Dogs with Healthy Gingiva, Gingivitis or Mild Periodontitis
Source: PLoS One. 2013 Dec 13;8(12):e83158. doi: 10.1371/journal.pone.0083158 (PMC3862762; doi:10.1371/journal.pone.0083158)
Supplement: Table S1 — The species that were grouped to genus/ clade level in Figure 1. The number of species grouped into each genus is also included. See figure S1 for a tree of these species. (DOC) [file pone.0083158.s002.doc]

Supplementary Table S1.

| **Species** | **Genus in Figure 1** | **# species grouped in genus** |
| --- | --- | --- |
| Filifactor villosus COT-031 | Filifactor | 3 |
| Peptostreptococcaceae bacterium COT-047 | Peptostreptococcaceae clade A | 2 |
| Frigovirgula sp. COT-007 clone OB027 | Frigovirgula | 5 |
| Peptostreptococcaceae bacterium COT-004 | Peptostreptococcaceae clade B | 5 |
| Peptostreptococcus sp. COT-033 | Peptostreptococcus | 1 |
| Peptostreptococcaceae bacterium COT-068 | Peptostreptococcaceae clade C | 8 |
| Peptostreptococcaceae bacterium COT-019 | Peptostreptococcaceae clade D | 6 |
| Parvimonas sp. COT-035 | Parvimonas | 3 |
| Peptostreptococcaceae bacterium COT-030 | Peptostreptococcaceae clade E | 1 |
| Helcococcus sp. COT-069 | Helcococcus | 2 |
| Peptoniphilus sp. COT-304 | Peptoniphilus | 1 |
| Peptostreptococcaceae bacterium COT-077 | Peptostreptococcaceae clade F | 1 |
| Tissierella sp. COT-029 | Tissierella | 1 |
| Clostridiales bacterium COT-028 | Clostridiales clade A | 6 |
| Lachnospiraceae bacterium COT-036 | Lachnospiraceae clade A | 4 |
| Blautia sp. COT-337 | Blautia | 1 |
| Catonella sp. COT-158 | Catonella | 4 |
| Lachnospiraceae bacterium COT-024 | Lachnospiraceae clade B | 5 |
| Clostridiales bacterium COT-388 | Clostridiales clade B | 5 |
| Peptococcus sp. COT-044 | Peptococcus | 1 |
| Dialister invisus COT-043 | Dialister | 1 |
| Schwartzia sp. COT-063 | Schwartzia | 2 |
| Selenomonas sp. COT-167 | Selenomonas | 2 |
| Streptococcus sp. COT-279 | Streptococcus | 7 |
| unclassified Aerococcaceae HM277601 | unclassified Aerococcaceae | 1 |
| Globicatella sp. COT-107 | Globicatella | 1 |
| Granulicatella sp. COT-095 | Granulicatella | 1 |
| Abiotrophia sp. COT-229 | Abiotrophia | 1 |
| Gemella palaticanis COT-089 | Gemella | 1 |
| Erysipelotrichaceae bacterium COT-311 | Erysipelotrichaceae | 5 |
| unclassified Bacteria HM333100 | unclassified Bacteria | 1 |
| Acholeplasmatales bacterium COT-375 | Acholeplasmatales | 2 |
| uncultured Leptotrichiaceae AJ289180 | Leptotrichiaceae clade A | 1 |
| Streptobacillus sp. COT-370 | Streptobacillus | 1 |
| Leptotrichia sp. COT-345 | Leptotrichia | 2 |
| Fusobacterium sp. COT-189 | Fusobacterium | 4 |
| Actinomyces sp. COT-083 | Actinomyces clade A | 8 |
| Actinomyces canis COT-409 | Actinomyces clade B | 8 |
| Actinomyces sp. COT-252 | Actinomyces clade C | 2 |
| Corynebacterium freiburgense COT-403 | Corynebacterium | 6 |
| Propionibacterium sp. COT-300 | Propionibacterium | 8 |
| Leucobacter sp. COT-288 | Leucobacter | 2 |
| uncultured Euzebya HM312659 | Euzebya | 3 |
| Atopobium sp. COT-418 | Atopobium | 1 |
| Treponema sp. COT-233 | Treponema clade A | 7 |
| Treponema sp. COT-356 clone 1Y140 | Treponema clade B | 3 |
| Treponema denticola COT-197 | Treponema clade C | 6 |
| Spirochaeta sp. COT-314 | Spirochaeta | 1 |
| unclassified Anaerolineaceae FN563258 | Anaerolineaceae | 1 |
| Chloroflexi bacterium COT-306 | Chloroflexi | 1 |
| Synergistales bacterium COT-138 | Synergistales clade A | 4 |
| Synergistales bacterium COT-178 | Synergistales clade B | 1 |
| Conchiformibius sp. COT-286 | Conchiformibius | 6 |
| Neisseria shayeganii COT-090 | Neisseria | 7 |
| Eikenella sp. COT-049 | Eikenella | 1 |
| Aquaspirillum sp. COT-091 | Aquaspirillum | 1 |
| Propionivibrio sp. COT-223 | Propionivibrio | 1 |
| Brachymonas sp. COT-015 | Brachymonas | 2 |
| Comamonas sp. COT-270 | Comamonas | 1 |
| unclassified Comamonadaceae HM297266 | unclassified Comamonadaceae | 1 |
| Ottowia sp. COT-014 | Ottowia | 1 |
| Xenophilus sp. COT-264 | Xenophilus | 2 |
| Lautropia sp. COT-175 | Lautropia | 2 |
| Pasteurella dagmatis COT-092 | Pasteurella | 3 |
| Pasteurellaceae bacterium COT-272 clone ZR110 | Pasteurellaceae clade A | 2 |
| Pasteurellaceae bacterium COT-080 | Pasteurellaceae clade B | 5 |
| Escherichia coli COT-277 | Escherichia | 1 |
| Moraxella sp. COT-396 | Moraxella | 3 |
| Cardiobacterium sp. COT-177 | Cardiobacterium | 4 |
| uncultured Xanthomonadaceae bacterium HM438438 | Xanthomonadaceae | 1 |
| Stenotrophomonas sp. COT-224 | Stenotrophomonas | 1 |
| Wolinella sp. COT-173 | Wolinella | 1 |
| Campylobacter sp. COT-011 | Campylobacter | 1 |
| Desulfovibrionales bacterium COT-009 | Desulfovibrionales | 1 |
| Desulfomicrobium orale COT-008 | Desulfomicrobium | 1 |
| Desulfovibrio sp. COT-070 | Desulfovibrio | 1 |
| Desulfobulbus sp. COT-078 | Desulfobulbus | 1 |
| Porphyromonas cangingivalis COT-109 | Porphyromonas clade A | 11 |
| Porphyromonas sp. COT-290 | Porphyromonas clade B | 7 |
| Tannerella forsythia COT-023 | Tannerella | 1 |
| Paludibacter sp. COT-384 | Paludibacter | 1 |
| Porphyromonadaceae bacterium COT-184 | Porphyromonas clade C | 1 |
| Proteiniphilum sp. COT-385 | Proteiniphilum | 1 |
| Prevotella sp. COT-298 | Prevotella | 8 |
| Bacteroides sp. COT-040 | Bacteroides | 3 |
| Odoribacter denticanis COT-084 | Odoribacter | 1 |
| Bacteroidia bacterium COT-187 | Bacteroidia clade A | 2 |
| unclassified Bacteroidales EU656097 | Bacteroidia clade B | 2 |
| Capnocytophaga cynodegmi COT-254 | Capnocytophaga clade A | 6 |
| Capnocytophaga sp. COT-339 | Capnocytophaga clade B | 2 |
| Cloacibacterium sp. COT-320 | Cloacibacterium | 1 |
| Bergeyella zoohelcum COT-186 | Bergeyella | 1 |
| Chlorobi bacterium COT-046 clone OB015 | Chlorobi | 4 |
| uncultured Elusimicrobium AB299538 | Elusimicrobium | 1 |
| uncultured TM7 DQ815554 | TM7 clade A | 3 |
| TM7 phylum sp. COT-251 | TM7 clade B | 2 |
| TM7 phylum sp. COT-305 | TM7 clade C | 2 |
| uncultured SR1 EU681994 | SR1 clade A | 1 |
| SR1 bacterium COT-369 | SR1 clade B | 2 |
