# Supplementary material for: A Cross-Sectional Survey of Bacterial Species in Plaque from Client Owned Dogs with Healthy Gingiva, Gingivitis or Mild Periodontitis
Source: PLoS One. 2013 Dec 13;8(12):e83158. doi: 10.1371/journal.pone.0083158 (PMC3862762; doi:10.1371/journal.pone.0083158)
Supplement: Table S2 — Summary of OTUs significantly associated with health (H), gingivitis (G) or mild periodontitis (PD1). (DOC) [file pone.0083158.s003.doc]

Supplementary Table S2.

| **Species** | % identity | **Average Proportion** | | | **Fold Change** | | | **Pair-wise *p*-value between health states** | | |
| --- | --- | --- | --- | --- | --- | --- | --- | --- | --- | --- |
|  |  | **H** | **G** | **PD1** | **H/G** | **H/ PD1** | **G/ PD1** | **H v G** | **H v PD1** | **G v PD1** |
| *uncultured Actinomyces sp. GU227175* | 96.9 | 0.00% | 0.02% | 0.10% | 0.10 | 0 | 0.1 | 0.1255 | 0.0026 | <0.001 |
| *Bacteroides sp. COT-040* | 100.0 | 0.86% | 0.50% | 0.30% | 1.60 | 3.2 | 2.1 | 0.0231 | <0.001 | 0.0057 |
| *Odoribacter denticanis COT-084* | 99.7 | 0.02% | 0.10% | 0.20% | 0.20 | 0.1 | 0.7 | <0.001 | <0.001 | 0.1065 |
| *Bergeyella zoohelcum COT-186* | 99.1 | 5.48% | 2.60% | 0.80% | 2.10 | 6.5 | 3.1 | 0.001 | <0.001 | 0.002 |
| *Capnocytophaga canimorsus COT-235* | 100.0 | 0.65% | 0.40% | 0.10% | 1.50 | 5.5 | 3.7 | 0.0762 | <0.001 | <0.001 |
| *Capnocytophaga cynodegmi COT-254* | 100.0 | 1.24% | 1.00% | 0.30% | 1.20 | 3.6 | 3 | 0.3334 | <0.001 | <0.001 |
| *Capnocytophaga sp. COT-295* | 100.0 | 0.84% | 0.20% | 0.07% | 5.30 | 11.9 | 2.3 | <0.001 | <0.001 | 0.1246 |
| *Capnocytophaga sp. COT-339* | 100.0 | 2.96% | 0.40% | 0.40% | 6.70 | 7.2 | 1.1 | <0.001 | <0.001 | 0.8919 |
| *Cloacisp. sp. COT-320* | 99.1 | 0.20% | 0.10% | 0.03% | 2.10 | 7.7 | 3.6 | 0.0021 | <0.001 | 0.0016 |
| *Chloroflexi sp. COT-306* | 100.0 | 0.21% | 0.40% | 0.90% | 0.50 | 0.2 | 0.5 | 0.0206 | <0.001 | <0.001 |
| *Helcococcus sp. COT-069* | 99.4 | 0.10% | 0.50% | 1.00% | 0.20 | 0.1 | 0.5 | <0.001 | <0.001 | 0.0049 |
| *Lachnospiraceae sp. COT-036* | 100.0 | 0.36% | 0.60% | 1.70% | 0.60 | 0.2 | 0.4 | 0.051 | <0.001 | <0.001 |
| *Lachnospiraceae sp. COT-106* | 99.7 | 0.30% | 0.20% | 0.06% | 1.20 | 5.1 | 4.2 | 0.2696 | <0.001 | <0.001 |
| *Peptococcus sp. COT-044* | 99.7 | 0.27% | 0.50% | 1.20% | 0.50 | 0.2 | 0.4 | 0.0479 | <0.001 | <0.001 |
| *Filifactor alocis COT-001* | 99.7 | 0.03% | 0.05% | 0.30% | 0.50 | 0.1 | 0.2 | 0.2332 | <0.001 | <0.001 |
| *Filifactor sp. COT-164* | 100.0 | 0.04% | 0.10% | 0.50% | 0.30 | 0.1 | 0.2 | 0.034 | <0.001 | <0.001 |
| *Peptostreptococcaceae sp. COT-004* | 100.0 | 1.09% | 1.50% | 3.50% | 0.70 | 0.3 | 0.4 | 0.2292 | <0.001 | <0.001 |
| *Peptostreptococcaceae sp. COT-019* | 100.0 | 0.26% | 0.50% | 1.20% | 0.50 | 0.2 | 0.4 | 0.0221 | <0.001 | <0.001 |
| *Peptostreptococcaceae sp. COT-021* | 100.0 | 0.37% | 0.40% | 0.90% | 1.00 | 0.4 | 0.5 | 0.8728 | <0.001 | <0.001 |
| *Peptostreptococcaceae sp. COT-067* | 99.7 | 0.01% | 0.06% | 0.10% | 0.10 | 0 | 0.4 | 0.0014 | <0.001 | <0.001 |
| *Peptostreptococcaceae sp. COT-155* | 99.7 | 0.01% | 0.05% | 0.10% | 0.20 | 0.1 | 0.4 | 0.0017 | <0.001 | <0.001 |
| *Peptostreptococcaceae sp. COT-030* | 100.0 | 0.04% | 0.40% | 1.10% | 0.10 | 0 | 0.4 | 0.0024 | <0.001 | <0.001 |
| *Peptostreptococcaceae sp. COT-077* | 100.0 | 0.06% | 0.80% | 2.70% | 0.10 | 0 | 0.3 | 0.0059 | <0.001 | <0.001 |
| *Clostridiales sp. COT-027* | 99.7 | 0.07% | 0.30% | 0.70% | 0.30 | 0.1 | 0.4 | 0.0017 | <0.001 | <0.001 |
| *Clostridiales sp. COT-028* | 100.0 | 0.26% | 1.10% | 2.20% | 0.20 | 0.1 | 0.5 | <0.001 | <0.001 | <0.001 |
| *Clostridiales sp. COT-388* | 100.0 | 0.02% | 0.20% | 0.30% | 0.10 | 0.1 | 0.7 | <0.001 | <0.001 | 0.0448 |
| *Schwartzia sp. COT-063* | 99.7 | 0.02% | 0.30% | 0.50% | 0.10 | 0.1 | 0.6 | <0.001 | <0.001 | 0.0485 |
| *Erysipelotrichaceae sp. COT-302* | 99.7 | 0.05% | 0.10% | 0.40% | 0.40 | 0.1 | 0.3 | 0.0573 | <0.001 | <0.001 |
| *Lautropia sp. COT-175* | 99.7 | 0.96% | 0.70% | 0.30% | 1.40 | 3.2 | 2.2 | 0.0678 | <0.001 | 0.0033 |
| *Brachymonas sp. COT-015* | 99.4 | 0.54% | 0.40% | 0.10% | 1.40 | 3.6 | 2.6 | 0.0538 | <0.001 | <0.001 |
| *Comamonas sp. COT-270* | 100.0 | 0.15% | 0.04% | 0.02% | 3.30 | 9.3 | 2.8 | <0.001 | <0.001 | 0.0305 |
| *Neisseria shayeganii COT-090* | 100.0 | 3.28% | 1.70% | 0.30% | 1.90 | 9.5 | 5 | 0.0014 | <0.001 | <0.001 |
| *uncultured Neisseria FM872599* | 99.7 | 0.60% | 0.10% | 0.01% | 4.60 | 41.8 | 9.1 | <0.001 | <0.001 | 0.0271 |
| *Cardiosp. sp. COT-177* | 100.0 | 0.48% | 0.30% | 0.10% | 1.50 | 4.4 | 3 | 0.0506 | <0.001 | <0.001 |
| *Moraxella sp. COT-396* | 98.9 | 6.61% | 3.50% | 0.90% | 1.90 | 7.6 | 4 | <0.001 | <0.001 | <0.001 |
| *Pasteurellaceae sp. COT-080* | 100.0 | 2.37% | 1.20% | 0.30% | 2.00 | 9.3 | 4.6 | <0.001 | <0.001 | <0.001 |
| *Pasteurella canis COT-273* | 100.0 | 0.47% | 0.20% | 0.10% | 2.50 | 4.3 | 1.7 | <0.001 | <0.001 | 0.0609 |
| *Pasteurellaceae sp. COT-271* | 98.3 | 0.05% | 0.02% | 0.01% | 2.10 | 7.5 | 3.5 | 0.0068 | <0.001 | 0.007 |
| *Treponema denticola COT-197* | 99.7 | 0.28% | 0.50% | 0.70% | 0.60 | 0.4 | 0.7 | 0.0287 | <0.001 | 0.0327 |
| *Synergistales sp. COT-179* | 99.7 | 0.01% | 0.01% | 0.07% | 1.20 | 0.2 | 0.1 | 0.7476 | <0.001 | <0.001 |
| *Synergistales sp. COT-180* | 98.9 | 0.06% | 0.20% | 0.40% | 0.30 | 0.2 | 0.6 | <0.001 | <0.001 | 0.0282 |
| *Synergistales sp. COT-244* | 99.7 | 0.00% | 0.01% | 0.09% | 0.10 | 0 | 0.2 | 0.0905 | 0.0022 | <0.001 |
| *SR1 sp. COT-380* | 100.0 | 0.11% | 0.07% | 0.02% | 1.50 | 6.5 | 4.2 | 0.0559 | <0.001 | <0.001 |
| *Actinomyces suimastitidis AJ277385* | 96.9 | 0.02% | 0.04% | 0.20% | 0.50 | 0.1 | 0.2 | 0.2597 | <0.001 | <0.001 |
| *Porphyromonas cangingivalis COT-109* | 99.4 | 10.47% | 7.90% | 4.50% | 1.30 | 2.3 | 1.8 | 0.0753 | <0.001 | 0.004 |
| *Porphyromonas sp. COT-290* | 100.0 | 0.97% | 0.60% | 0.30% | 1.70 | 3.1 | 1.9 | 0.0139 | <0.001 | 0.016 |
| *uncultured Acetoanaerobium HM277905* | 99.7 | 0.10% | 0.20% | 0.60% | 0.60 | 0.2 | 0.3 | 0.2774 | <0.001 | <0.001 |
| *Peptostreptococcaceae sp. COT-068* | 99.7 | 0.04% | 0.30% | 0.50% | 0.20 | 0.1 | 0.6 | 0.0013 | <0.001 | 0.0209 |
| *uncultured Saccharofermentans EU381658* | 93.7 | 0.00% | 0.03% | 0.07% | 0.10 | 0.1 | 0.5 | 0.0052 | <0.001 | 0.0088 |
| *uncultured Flavonifractor FJ365194* | 98.9 | 0.02% | 0.10% | 0.20% | 0.20 | 0.1 | 0.6 | 0.0031 | <0.001 | 0.0339 |
| *uncultured TM7 FJ879268* | 97.4 | 0.00% | 0.01% | 0.05% | 0.20 | 0 | 0.2 | 0.1184 | <0.001 | <0.001 |
| *Conchiformibius steedae AF328156* | 99.4 | 0.28% | 0.06% | 0.01% | 4.40 | 19.7 | 4.5 | <0.001 | <0.001 | 0.0459 |
| *Synergistales sp. COT-138* | 99.7 | 0.01% | 0.07% | 0.09% | 0.20 | 0.2 | 0.7 | <0.001 | <0.001 | 0.1685 |
| *uncultured Actinomyces JF203363* | 96.0 | 0.04% | 0.04% | 0.20% | 0.90 | 0.3 | 0.3 | 0.8218 | <0.001 | <0.001 |
| *Capnocytophaga sp. COT-329* | 99.7 | 0.16% | 0.06% | 0.01% | 2.80 | 14.7 | 5.3 | <0.001 | <0.001 | 0.0075 |
| *Catonella sp. COT-257* | 100.0 | 0.14% | 0.08% | 0.04% | 1.80 | 3.5 | 2 | 0.0129 | <0.001 | 0.019 |
| *Peptostreptococcaceae sp. COT-129* | 100.0 | 0.01% | 0.06% | 0.10% | 0.10 | 0.1 | 0.5 | 0.0031 | <0.001 | 0.0133 |
| *Synergistales sp. COT-178* | 100.0 | 0.33% | 0.60% | 0.70% | 0.50 | 0.5 | 0.9 | 0.0026 | <0.001 | 0.3083 |
| *[Eusp.] nodatum COT-045* | 99.7 | 0.00% | 0.01% | 0.06% | 0.30 | 0 | 0.1 | 0.2527 | <0.001 | <0.001 |
| *uncultured Actinomyces HM336429* | 96.6 | 0.00% | 0.00% | 0.07% | 0.20 | 0 | 0 | 0.6623 | 0.0816 | 0.0033 |
| *Peptostreptococcaceae sp. COT-135* | 100.0 | 0.56% | 0.90% | 1.40% | 0.60 | 0.4 | 0.6 | 0.0483 | <0.001 | 0.021 |
| *Prevotella sp. COT-372* | 100.0 | 0.14% | 0.04% | 0.03% | 3.70 | 4.1 | 1.1 | <0.001 | <0.001 | 0.8096 |
| *Schwartzia sp. COT-213* | 98.9 | 0.00% | 0.00% | 0.09% | 1.20 | 0 | 0 | 0.9082 | <0.001 | <0.001 |
| *Erysipelotrichaceae sp. COT-381* | 99.7 | 0.03% | 0.10% | 0.20% | 0.40 | 0.2 | 0.5 | 0.023 | <0.001 | 0.0045 |
| *Treponema sp. COT-351* | 99.7 | 0.01% | 0.03% | 0.08% | 0.10 | 0.1 | 0.4 | 0.0102 | <0.001 | 0.0026 |
| *Bacteroidia sp. COT-187* | 99.7 | 0.02% | 0.06% | 0.20% | 0.40 | 0.1 | 0.3 | 0.1237 | <0.001 | <0.001 |
| *Parvimonas sp. COT-101* | 99.4 | 0.05% | 0.20% | 0.30% | 0.30 | 0.2 | 0.7 | 0.002 | <0.001 | 0.0682 |
| *Peptostreptococcaceae sp. COT-124* | 100.0 | 0.04% | 0.07% | 0.20% | 0.60 | 0.3 | 0.4 | 0.2099 | <0.001 | 0.0025 |
| *Lautropia sp. COT-060* | 100.0 | 0.66% | 0.30% | 0.20% | 2.10 | 2.9 | 1.4 | 0.0039 | <0.001 | 0.3209 |
| *Pasteurella dagmatis COT-092* | 100.0 | 0.50% | 0.60% | 0.10% | 0.80 | 3.5 | 4.4 | 0.3167 | <0.001 | <0.001 |
| *Corynesp. mustelae COT-419* | 100.0 | 0.53% | 0.70% | 0.20% | 0.80 | 2.6 | 3.3 | 0.2333 | 0.0026 | <0.001 |
| *Corynesp. canis COT-421* | 99.7 | 0.20% | 0.90% | 1.10% | 0.20 | 0.2 | 0.8 | <0.001 | <0.001 | 0.3379 |
| *Campylobacter sp. COT-011* | 100.0 | 0.82% | 0.80% | 0.50% | 1.00 | 1.7 | 1.7 | 0.9003 | 0.0017 | 0.002 |
| *Escherichia coli COT-277* | 99.7 | 0.10% | 0.06% | 0.03% | 1.80 | 3 | 1.7 | 0.0115 | <0.001 | 0.0687 |
| *Clostridiales sp. COT-038* | 100.0 | 0.04% | 0.10% | 0.40% | 0.30 | 0.1 | 0.3 | 0.0738 | <0.001 | <0.001 |
| *Acholeplasmatales sp. COT-375* | 100.0 | 0.01% | 0.02% | 0.06% | 0.70 | 0.2 | 0.3 | 0.3626 | <0.001 | <0.001 |
| *Globicatella sp. COT-107* | 100.0 | 0.88% | 0.40% | 0.30% | 2.20 | 2.7 | 1.2 | 0.0024 | <0.001 | 0.4696 |
| *Parvimonas sp. COT-035* | 99.4 | 0.04% | 0.20% | 0.90% | 0.20 | 0 | 0.2 | 0.0632 | <0.001 | <0.001 |
| *Moraxella sp. COT-328* | 100.0 | 0.27% | 0.20% | 0.02% | 1.40 | 14.9 | 10.6 | 0.2247 | <0.001 | <0.001 |
| *Lachnospiraceae sp. COT-161* | 99.4 | 0.08% | 0.10% | 0.04% | 0.70 | 2 | 2.8 | 0.1519 | 0.0161 | <0.001 |
| *Neisseria canis AY426973* | 100.0 | 0.00% | 0.03% | 0.00% | 0.00 | 0 | 11 | 0.377 | 0.5199 | <0.001 |
| *Desulfovibrionales sp. COT-009* | 100.0 | 0.02% | 0.20% | 0.20% | 0.10 | 0.1 | 1 | <0.001 | <0.001 | 0.8891 |
| *uncultured Capnocytophaga HM333068* | 100.0 | 0.04% | 0.10% | 0.01% | 0.40 | 3.2 | 7.3 | 0.0089 | 0.0185 | <0.001 |
| *Leptotrichia sp. COT-345* | 100.0 | 0.23% | 0.50% | 0.20% | 0.40 | 1.5 | 3.5 | 0.0031 | 0.2767 | <0.001 |
| *Aquaspirillum sp. COT-091* | 99.1 | 0.53% | 0.40% | 0.06% | 1.20 | 9.3 | 7.7 | 0.4646 | <0.001 | <0.001 |
| *Stenotrophomonas sp. COT-224* | 98.9 | 1.10% | 0.50% | 0.30% | 2.10 | 3.5 | 1.7 | 0.0045 | <0.001 | 0.1232 |
| *uncultured TM7 EF614904* | 98.0 | 0.02% | 0.05% | 0.06% | 0.40 | 0.3 | 0.8 | 0.0085 | <0.001 | 0.4009 |
| *Actinomyces hyovaginalis X69616* | 97.6 | 0.01% | 0.20% | 0.30% | 0.00 | 0 | 0.7 | 0.0133 | 0.006 | 0.1989 |
| *Clostridiales sp. COT-141* | 99.7 | 0.00% | 0.06% | 0.10% | 0.10 | 0 | 0.6 | 0.011 | 0.0025 | 0.0893 |
| *uncultured TM7 DQ815554* | 97.4 | 0.03% | 0.03% | 0.07% | 0.90 | 0.4 | 0.4 | 0.8595 | <0.001 | <0.001 |
